# Exploring school nurses' experiences of a metaverse-based infectious disease simulation training: qualitative study

**DOI:** 10.1080/17482631.2026.2647088

**Published:** 2026-03-24

**Authors:** Ihn Sook Jeong, Min Ji Kim, Youn Sun Hwang, Ju Hyun Kang, Mi Ok Kang, Jae Eun Cha, Young Kyung Seo

**Affiliations:** a College of Nursing, Pusan National University, Yangsan, Republic of Korea; b Sabryang Elementary School, Yangsan, Republic of Korea; c Department of Nursing, Dongseo University, Busan, Republic of Korea; d Institute of Convergence Education, Korea National University of Education, Cheongju, Republic of Korea; e Department of Nursing, Singyeongju University, Gyeongju, Republic of Korea; f Department of Education, Pusan National University, Busan, Republic of Korea; g Department of Nursing, Yeungjin University, Daegu, Republic of Korea

**Keywords:** Disease transmission, infectious, qualitative study, school health, simulation, virtual reality exposure therapy

## Abstract

**Purpose:**

This study aimed to explore school nurses’ experiences and perceptions of metaverse-based simulation training (MBST) for infectious disease, focusing on its educational value and applicability in real school settings.

**Methods:**

A qualitative study was conducted using focus group interviews with ten school nurses in Yangsan, Republic of Korea, between December 2024 and January 2025. Data were analyzed using an inductive approach informed by grounded theory, including open coding and constant comparison.

**Results:**

Four themes and ten categories emerged, including positive experiences of immersion and self-directed learning, negative experiences related to deviation from educational purpose, and factors influencing field application. Participants found MBST engaging and helpful for enhancing understanding, despite challenges such as technical issues, digital literacy gaps, and accessibility concerns.

**Conclusions:**

MBST shows strong potential to improve infectious disease preparedness among school nurses and other school personnel, highlighting the need for improved infrastructure and support.

## Introduction

Schools, where large numbers of students gather and interact closely, are susceptible to the rapid transmission of infectious diseases in the event of an outbreak. According to the 2024 Annual Report on Notified Infectious Disease in Korea, the number of infectious disease cases in Korea has increased by more than 54% compared to the previous year, which appears to be largely attributable to the increase in respiratory infections such as pertussis and scarlet fever, particularly among school-aged children (Korea Disease Control and Prevention Agency, 2025).

Supporting the physical, mental, and social health of school-aged children is essential to sustaining the long-term competitiveness of society. In response to these challenges, the Korea Ministry of Education has implemented national policies to strengthen infectious disease preparedness in schools, including the dissemination of prevention manuals and the mandate for annual simulation-based training (Ministry of Education, 2023).

Simulation-based training is designed to prepare for real-world infectious disease outbreaks through scenario-based exercises. However, in school settings, ensuring full participation from all involved staff members can be challenging, often resulting in training being conducted merely as a formality, limited to reading the scenario and reviewing individual roles (Korea Educational Environment Protection Agency, 2023). Therefore, there is a need to develop effective and practical strategies to implement infectious disease simulation training in school settings.

Simulation-based education has been widely recognised as an effective pedagogical approach that enhances learners' clinical reasoning, decision-making, and practical competencies by allowing repeated practice in realistic situations. Through experiential engagement rather than passive knowledge acquisition, simulation promotes deeper learning, increased confidence, and improved performance in real-life contexts. By simulating complex and unpredictable situations, it further supports active problem solving, reflective learning, and the integration of theoretical knowledge into practical action.

Recent technological advancements, accelerated by the COVID-19 pandemic, have contributed to making “untact” interactions a natural part of everyday life. As a result, digital transformation is accelerating across various sectors, including education, where nonface-to-face classes have been increasingly adopted in schools. The Korea Ministry of Education introduced innovative education reform with an emphasis on advancing digital literacy across all subjects, aiming to equip students with the foundational competencies necessary to actively participate in future social changes (Ministry of Education, 2021). Accordingly, digital literacy training for schoolteachers has been actively promoted, aiming to increase their capacity to apply digital tools in pedagogical instruction. Virtual reality, augmented reality, artificial intelligence (AI), and metaverse platforms are among the digital technologies increasingly utilised in educational sectors (Lee & Kim, 2025).

The metaverse has been increasingly recognised as a promising platform for simulation-based training because it provides immersive and interactive environments that extend beyond the limitations of traditional video conferencing or face-to-face classroom settings (Dionisio et al., 2013; Mystakidis, 2022). In the context of infectious disease management, metaverse-based environments allow participants to practice response protocols within safe virtual spaces that can simulate real-world school settings without exposure to actual infection risk. Emerging evidence suggests that avatar-based interaction and repetitive practice in metaverse environments may enhance learner engagement, motivation, and practical competency in health profession education (Ryu et al., 2024).

In this context, our research team developed and ran a short-term educational course to assist school nurses in developing bespoke metaverse-based simulation training (MBST) platforms for infectious disease management in schools in 2024 (Jeong et al., 2025). The five-day course consisted of three online sessions and two in-person sessions. During the course, using ZEP platform (ZEP Co., Ltd.), a freely accessible Korean metaverse tool, six scenario-based simulation platforms were developed: two for tuberculosis, two for influenza, one for chickenpox, and one for general respiratory infections. These platforms incorporate multimedia integration, real-time avatar interaction, games, missions, and scenario-based role-playing.

Before expanding their use to other schools, it is important to understand the experiences and perspectives of school nurses who lead infectious disease simulation training in schools to ensure the practical effectiveness and sustainability of the programme in real-world settings. Therefore, this study aimed to explore school nurses' experiences and perceptions of metaverse-based simulation training in depth, focusing on its educational value and applicability in school environments.

## Methods

### Study design

An exploratory descriptive qualitative research approach using focus group interview (FGI) was employed to seek a deeper understanding of school nurses' experiences with MBST. FGI allows participants to interact and build on each other's experiences, leading to richer and more nuanced insights (Creswell & Poth, 2018). The study followed the Standards for Reporting Qualitative Research (SRQR) guidelines (O'Brien et al., 2014).

### Sample and recruitment

To recruit study participants, the purpose of the study was first explained to the president of the school nurses' association in Yangsan city, and a recruitment notice was then posted on the association's website. For the potential participants who expressed the willingness to participate, the researcher sent an information sheet and consent form via e-mail and explained the purpose and procedures of the study by phone when required. Ten school nurses who met the inclusion criteria agreed to participate, and their detailed characteristics are presented in the Results section. The sample size of 10 was manageable, generated sufficient qualitative data, and was feasible in terms of access.

### Data collection

Prior to the interview, the participants were provided with access to six metaverse-based simulation training platforms to allow them to explore in advance. The interviews were conducted online using Zoom application in two separate sessions, each consisting of five participants on 30 December 2024, and 5 January 2025. The interview schedules and Zoom access links were provided via email or telephone. Three researchers conducted both interviews. At the outset, the participants were informed about the role of each researcher and that the interviews would be recorded. A brief self-introduction session was then held to help participants feel more comfortable and encourage open discussion. Afterwards, one researcher introduced the tuberculosis platforms to the participants and guided them to explore by serving as a moderator. While the participants engaged with the developed platforms, the other two researchers observed the participants' responses.

Upon completion, the participants were encouraged to share their experiences. To gain deeper insight into the participants' experiences and perceptions, the researchers employed semi-structured, open-ended questions and used probing questions when needed to clarify the participants' statements. The interviews were structured around the following three overarching research questions:

1. What is your experience of using metaverse-based simulation training, compared to existing face-to-face simulation training?
2. What are the anticipated effects and potential limitations of applying them in real school settings?
3. What are the essential support systems and directions for improvement to facilitate actual implementation?

Each interview session lasted approximately 2–2.5 h, including platform exploration. The Zoom recordings were converted into an audio file and transcribed using an AI transcription tool on the same day. To ensure the rigour of the analysis, all five researchers cross-checked the transcribed files against the original recordings. For the second interview, the same set of questions was used. Data saturation was confirmed during the second session when no new themes or significant information emerged from the participants' discussions, indicating that the sample size of ten was sufficient for this qualitative exploration.

### Data analysis

The data were analysed using an inductive approach informed by grounded theory, specifically the procedures of open coding. First, the collected data were transcribed, and each participant's statements were labelled with identifiers (A–J) to maintain anonymity. The data were stored on password-protected personal computers, and the transcripts were cross-checked with audio recordings to ensure accuracy.

The transcripts were read repeatedly to identify meaning units. Through open coding and constant comparison, statements with similar meanings were grouped and labelled as initial codes. The codes were continuously compared and integrated into higher-level conceptual categories to derive overarching themes. Five researchers conducted the analysis collaboratively. At each stage, the codes and emerging categories were reviewed and refined through consensus-building. Discrepancies in interpretation were resolved through discussion.

### Rigour

This study was guided by a methodology to ensure the reliability and validity of qualitative research (Creswell & Poth, 2018). The research team consisted of current and former school nurses, along with qualitative researchers, who combined a practical understanding of the school setting with expertise in qualitative research methodology. Practical field experience and theoretical insights were incorporated into the data collection and integrated during the analysis process. The reliability and validity of the data interpretation were ensured through continuous discussion among the researchers. The recorded data were transcribed promptly following each interview and reviewed repeatedly to confirm key points, and the accuracy and meaning of the statements were verified through a review process. In the data analysis process, the criteria and process for extracting themes were documented so that other researchers could draw similar conclusions when interpreting the same data. In addition, to verify that the analysed content was consistent with the intentions of the participants, a member-checking procedure was conducted. Summarised analyses were shared with some participants, and their opinions were reflected in the final analysis.

### Ethical considerations

Ethical approval was obtained from the Institutional Review Board (IRB) of P University (2024_199_HR). The participants were provided with thorough information in advance regarding the purpose, procedures and recording of interview, and verbal and written information was obtained. They were informed that the collected data would be kept strictly anonymous and would not be used for any purposes other than the study. In addition, they were made aware of their right to withdraw from participation at any time during the study process without any disadvantages. The interview schedules flexibly prioritised the participants' availability and convenience.

Following the interview, a small token of appreciation (a gift voucher worth approximately 15 USD) was provided to each participant. This was intended as a standard compensation for their time and contribution to the study rather than an incentive to influence participation or bias the results. The amount was determined in accordance with the IRB guidelines of P university to ensure that it did not constitute an undue inducement. All participants were adults, and written informed consent for participation was obtained prior to the interview. No minors were included in this study.

## Results

The general characteristics of the ten participants are as follows: eight were working in elementary schools, and two were working in middle schools. The mean age of the participants was 31.9 years, and their average tenure as a school nurse was 5.7 years (Table I). This sample represents early- to mid-career school nurses with practical experience in school health settings.

**Table I.** General characteristics of participants.

Session	Subject	Sex	Age	Type of school	Position	Working years
1	A	F	34	Elementary	School nurse	8
	B	F	27	Elementary	School nurse	4
	C	F	29	Elementary	School nurse	4
	D	F	29	Elementary	School nurse	4
	E	F	38	Middle school	School nurse	7
2	F	F	38	Elementary	School nurse	6
	G	F	27	Elementary	School nurse	4
	H	F	29	Elementary	School nurse	5
	I	F	29	Elementary	School nurse	6
	J	F	39	Middle school	School nurse	9

F = Female.

From the participants' experiences of metaverse-based simulation training, four themes emerged: (1) positive experiences of immersion and self-directed learning, (2) negative experiences reflecting a deviation from the educational purpose, (3) barriers to field application, and (4) facilitating factors for practical application (Table II).

**Table II.** Themes from participants' experiences with metaverse-based infectious disease simulation training.

Theme	Categories
1. Positive experiences of immersion and self-directed learning	1) Immersive learning experience 2) Promoting self-directed participation
2. Negative experiences concerning a deviation from the educational purpose	1) Potential risks of overemphasising engagement 2) Limitations of the simulation scenario
3. Barriers to field application	1) Challenges in developing a practice-based programme 2) Environmental constraints in programme implementation 3) Insufficient support system for programme operation 4) Resistance from programme participants
4. Facilitating factors for field application	1) School nurses' willingness and expectations for utilisation 2) Strengthening school nurses' competencies for implementation

### Positive experiences of immersion and self-directed learning

The participants agreed that the MBST is highly engaging and immersive, effectively encouraging active participation. Unlike current face-to-face simulation training, which is highly dependent on script reading, the MBST, which integrates visual and experiential components, was positively evaluated for enhancing learners' immersion and promoting self-directed participation.

#### Immersive learning experience

The participants described that the visually presented and gamified content in MBST was stimulating and immersive and actively moving through the virtual space and experiencing each part of the training, which made the programme particularly effective.

*“The biggest difference is that this method really helps attract our interest and make us to focus. That’s the main reason I tried using the metaverse technologies in class. So, I think if schools start using this, trainings that used to be boring and something just had to get through can now be a bit more fun and engaging. Plus, the key points covered in the quiz could actually help remember important content in a more meaningful way.”* (Participant E)

*“Well, first of all, I find it really fun and cute, so it definitely makes me really interested*….. *So I actually think doing it this way might help people focus more, and since it’s much more visual, it could be a bit more effective.”* (Participant H)

#### Promoting self-directed participation

Participants noted that the use of quizzes, games, and visually intuitive features in the MBST assisted to identify areas of uncertainty, facilitated self-directed learning and supported repetitive review. In addition, the MBST encouraged them to take the initiative in their own roles.

*“There are things you already know and things you don’t. There are a lot to take in, so you can go back and check the parts you didn’t get. I think that’s what makes it better. (…) You can just look it up and go over it yourself.”* (Participant C)

### Negative experiences concerning a deviation from the educational purpose

While MBST has the advantage of promoting learner engagement by stimulating interest and inducing immersion, the participants also expressed concerns that entertaining elements might dilute the core purpose of the training, emphasising the need to maintain a balanced and purposeful learning experience. Specifically, the participants expressed concern that learners might focus more on completing game-like tasks, such as collecting stamps or moving quickly between virtual spaces, rather than fully engaging with the educational content and understanding the overall response process to infectious disease situations.

#### Potential risks of overemphasising engagement

Some participants acknowledged that while stimulating interest is certainly a strength, gamified features such as stamp collection and overly entertainment-oriented elements could undermine the primary purpose of simulation training. They pointed out that without a well-structured simulation scenario and systematic content organisation, participants may struggle to grasp the overall context. They suggested that a clearly organised scenario should be provided, along with specific guidance on each training stages. The participants suggested that setting a minimum time for learners to remain in each virtual space could help ensure adequate knowledge acquisition.

*“It’s great that it’s fun. That’s definitely a plus. But I feel like for some people, that fun could actually take away from the real purpose. They might just focus on getting the stamps and skip around to different places without really paying attention. (…) So I think it’d be nice if there was some kind of system where you have to stay in each area for like 20 or 30 seconds, just to make sure you actually look around and take it all in.”* (Participant C)

#### Limitations of simulation scenario

The participants emphasised the importance of coordinated collaboration among various personnel, including school nurses, grade-level coordinators, administrators, and homeroom teachers, during infectious disease responses. They suggested that incorporating scenarios involving visits from epidemiological investigation teams could enhance the realism and depth of the training. For example, the scenario should specifically include the roles of administrative staff, the need for consistency of information, improved visualisation of movement paths, step-by-step guidance throughout the simulation, and tailored scenarios for different target groups, including students and school staff.

*“...Even though school nurses are the ones in charge, honestly, everyone staff members have some kind of role. (…) So I was thinking it’d be great if the metaverse training also gave other school staff a chance to talk about their challenges. That might really get everyone more involved. (…) When there's an actual outbreak and the health centre comes in to do the epidemiological investigation. (…) If we could include more of that kind of scenario in the training, I think it would make it feel way more realistic and hands-on.”* (Participant I)

### Barriers to field application

While participants recognised the educational value of MBST, they also identified several structural and practical challenges that must be addressed for successful implementation in real school settings. Key concerns included the significant time and technical expertise required to develop the programme, poor network infrastructure and disparities in device performance, lack of personnel to support programme operation, and passive attitudes of teachers and staff. These factors highlight the need for ongoing institutional support and a well-established infrastructure to effectively integrate advanced educational technologies into the school environment.

#### Challenges in developing a practice-based programme

The participants perceived that developing MBST platform themselves for the school as impractical because of technical burdens and time constraints. In addition, the lack of incentives contributed to passive attitudes toward platform creation.

*“I actually tried making a metaverse-based lesson for my students. I added items one by one, inserted links, hid clues, and even tested out different portal setups. But honestly, it just took way too much time to build everything by myself. (…) I mean, spending months making a whole map for just one simulation training a year?”* (Participant J)

*“I tried ZEPETO before……The controls are kind of complicated, and even just thinking about making something on it seems like it would take a lot of time. I think using ZEPETO could make things feel more realistic and fun, but it might be a little challenging.”* (Participant G)

*“There’s just no real reward for trying something new or creative like this. I already have so much work to do and making something like this doesn’t mean people will think I’m doing a better job. I really don’t see the point. There’s just no motivation.”* (Participant I)

#### Environmental constraints in programme implementation

From an environmental standpoint, the participants identified that unstable Wi-Fi connections, disparities in personal device performance, and variability in access to digital devices could lead to delays or technical errors during MBST.

*“Everyone’s using different kinds of digital devices, right? And sometimes the way you connect can vary depending on the device. So, explaining all that could take quite a bit of time. … Also, if the internet connection isn’t stable, it can get disconnected, and then you have to log back in again.”* (Participant D)

#### Insufficient support system for programme operation

The participants asserted the limitations of relying on a single school nurse to support a large number of participants. Additionally, they expressed concern that if MBST were expanded to students, varying levels of digital literacy and the time required to teach platform use might divert attention from the core goals of health education.

*“If someone gets lost along the way, I feel like I’d be the only one helping them. and honestly, with the time limit, that might be a bit hard. We have a set schedule, and with almost 90 staff members altogether, it’d be really hard to support everyone one by one.”* (Participant B)

*“What really gets tricky about using edtech in middle school is that the classes are all split by subject, right? So, if I try to use a platform that requires a lot of technical know-how during my health class, I start wondering ‘Am I teaching health or computer science?’”* (Participant J)

#### Resistance from programme participants

The participants mentioned that resistance to implementing new programmes may vary depending on the school's organisational culture and the attitudes of administrators.

*“If our school gives this a try, I think a lot of people will push back. Even though everyone says AI and edtech matter, most just stick with what they know. So I feel like at first, a lot of people would be asking, ‘Why do we have to do it this way?*’” (Participant F)

*“It really depends on the school culture. Some might go, ‘Ugh, this sounds like a hassle,’ and not care. But if the principal’s on board and says, ‘Let’s try it,’ the vibe could shift. Honestly, hard to tell how it'll go.*” (Participant E)

### Facilitating factors for field application

This category captures the key factors in the practical implementation of metaverse-based simulation training in school settings. The participants emphasised that successful field application depends not only on technological readiness but also on school nurses' willingness, professional competencies, and institutional support. These findings highlight the conditions necessary for translating MBST into sustainable practice in real school environments.

#### School nurses' willingness and expectations for utilisation

While the participants openly admitted the burden associated with creating metaverse developed platforms themselves, they also expressed a strong willingness to actively utilise them. Their responses reflected an increased awareness of creative teaching methods, a deeper appreciation of the educational values, a recognition of new directions for health education, and a sense of anticipation regarding practical implementation.

*“Honestly, I caught myself thinking, ‘Why didn’t I think of using the metaverse like this?’ I kept wondering why I was so set on doing things the same old way, and why I hadn’t tried to think outside the box. It really made me reflect on that.”* (Participant C)

*“Whenever I took AI-related trainings, I’d just think, ‘ the world’s really changing,’ and leave it at that. I never thought of using it to my health classes. But through this experience, I realised, I could use AI to make infectious disease training more fund and engaging. That really clicked for me.”* (Participant B)

#### Strengthening school nurses' competencies for implementation

While school nurses are increasingly aware of the need for digital competencies, there is a shared perception that the technical learning curve and complexity of content creation present significant barriers to entry. Nevertheless, the school nurses’ sense of professional identity and calling served as key motivators for developing their competencies. In addition, effective use of the MBST requires not only technical skills but also a comprehensive set of professional competencies, including socio-emotional leadership, data privacy, and ethical and legal knowledge related to adapting cyberspace.

*“These days, with AI becoming a bigger part of education, there's a lot of talk about building social-emotional skills. And honestly, I think we also need to understand the legal stuff around personal data. Plus, there’s the tech side we’ve got to keep up with that too. And for school nurses, I feel like communication is something we tend to overlook, but it's definitely something we need to work on more.”* (Participant A)

## Discussion

Through focus group interviews, the participants discussed their positive learning experiences, expressed concerns about the potential dilution of educational objectives due to an overemphasis on engagement, and identified both barriers to and facilitating factors for field application. The findings revealed that, for the programme to be effectively utilised in real-world settings, not only are technological and environmental infrastructures necessary but also the enhancement of digital competencies, organisational collaboration, and the design of content aligned with educational goals.

The school nurses who participated in this study evaluated MBST as more effective in promoting engagement, compared to the conventional face-to-face training. In particular, the ability to explore virtual spaces, engage in quizzes, and interact with avatars allows visual and experiential learning, which encourages active and immersive participation in the training process. This immersive experience contributes to self-directed learning and is closely related to Bandura's (1982) self-efficacy theory.

Bandura (1982) defined self-efficacy as an individual's belief in their ability to successfully perform a specific task, emphasising its direct influence on motivation, emotional responses, and behavioural patterns. In this study, the participants reported a sense of self-efficacy as they actively engaged with the simulation scenarios, accessed relevant information through various means, and navigated the problem-solving processes. In particular, the immediate feedback provided during problem-solving tasks aligns closely with the core principles of Bandura's self-efficacy theory.

Furthermore, the findings of this study can also be interpreted through the lens of constructivist learning theory. Constructivist theory proposes that learners are not passive recipients of information but rather active constructors of meaning through social interaction and engagement in authentic contexts (Jonassen, 1991).

Instead of merely reading through the scenario, they are able to internalise infectious disease response procedures by walking through virtual spaces, observing situations, and making decisions using avatars. Through this process, they engage in meaningful learning grounded in their own experiences.

Previous studies on infection control education using virtual reality (VR) have also demonstrated that providing a sense of realism and interactivity enhances learner immersion (Kim, 2023). However, metaverse-based environments not only offer immersive elements but also strengthen self-directed learning by allowing learners to choose diverse learning paths and explore information at their own pace. From this perspective, metaverse-based programmes can serve as an effective educational strategy not only for infectious disease simulation training but also for broader health education among students. Especially in complex situations such as managing infectious diseases in school settings, situational judgement and practical competence are essential. Thus, immersive simulations that effectively train these skills can be valuable tools in real-world educational settings.

Meanwhile, some participants expressed concerns that an excessive focus on student engagement might dilute fundamental educational purposes. Gamified missions might cause learners to focus primarily on task completion rather than on engaging with the underlying educational content, which may undermine learning and the development of practical infectious disease response skills. They emphasised the need to maintain a balance between original goals and motivational elements.

This finding aligns with previous research on the dual effects of game-based learning, which, while incorporating game elements such as points, avatars, and immediate feedback to enhance learner motivation, also presents challenges in preserving the core intent of instruction (Alsawaier, 2018). At the same time, there are concerns that learners may prioritise external rewards over essential learning goals or become engrossed in game mechanics at the expense of educational outcomes (Hanus & Fox, 2015).

This highlights the importance of setting clear learning objectives and providing appropriate feedback, the core principles of effective instructional design (Merrill, 2017). Without a clear understanding of what knowledge is to be acquired and why specific activities are being undertaken, learners' immersion may not lead to meaningful learning but instead remain a superficial experience.

Although the metaverse environment offers a flexible structure that allows learners to explore and interact freely, the greater autonomy, the more critical it becomes to provide clear guidance on the direction and objectives of the simulation training.

Several participants in this study also mentioned the need for supplementary measures such as setting a minimum stay time in each virtual space, designing a scenario-based, step-by-step learning flow, and providing summary guides to support effective learning. Therefore, for MBST to achieve its intended educational goals, the content must go beyond capturing interest and be supported by a structural design. While visual and gamified elements that stimulate immersion can increase interest and behavioural engagement, they must ultimately be integrated in a way that promotes learners' cognitive engagement and supports the achievement of training goals (Taşkın & Kılıç Çakmak, 2023).

Considering the applicability of MBST in real school settings, several practical constraints have been identified. In particular, the lack of technological infrastructure, disparities in digital accessibility among users, and connectivity issues caused by the use of diverse devices have emerged as major barriers. Previous studies have pointed out that the digital divide in educational settings extends beyond mere device ownership to include disparities in digital literacy, technical support, and overall accessibility (Lim et al., 2024; Yoon et al., 2021). The effective use of metaverse platforms in group settings requires a high-performance network environment and a certain level of digital proficiency.

Therefore, differences in digital skills among simulation participants may lead to disparities in training effectiveness. To address these challenges, it is essential to provide participants with sufficient guidance and opportunities for practice prior to the implementation. This includes providing clear access manuals, securing technical support personnel, and conducting pre-assessments of device and network environments. In addition, relying on a single school nurse to implement the programme was considered insufficient to support large-scale participation. The participants emphasised the need for systematic preparation at the organisational level to facilitate collaborative engagement among all staff members.

For the training to be implemented effectively, it is essential to ensure role sharing and collaboration among school members, active involvement of administrators and administrative staff, and a shared understanding of the educational purpose among all faculty members. The participants in this study suggested that incorporating scenarios that reflect actual collaboration structures and role-based participation could enhance the effectiveness of the training. These needs extend beyond programme design and call for a shift in school organisational culture and a systemic approach. A support system led by school administrators, provision of digital literacy training, and promoting awareness of metaverse applications are key factors in enhancing the acceptance and applicability of the training. This suggests that a successful MBST requires not only the adoption of technology but also comprehensive institutional support, including staff development and administrative infrastructure. Fostering a positive atmosphere through school leadership was emphasised to improve infrastructure, and support systems require strong collaboration within the school community.

Finally, many participants expressed that developing an MBST platform themselves posed significant burdens in terms of time, expertise, and motivation. The professionalism of school nurses extends beyond the delivery of health-related content and encompasses the ability to adapt to evolving educational environments and to design innovative teaching and learning strategies (Priestley & Drew, 2019). This suggests that while school nurses may be accustomed to acting as consumers of educational content, they are not yet adequately prepared to take on the role of content creators. In particular, the metaverse environment requires a level of expertise that goes beyond basic content editing. It involves skills such as virtual space planning, designing digital interactions, and solving technical problems. Therefore, to ensure the sustainability and scalability of MBST, there is a need to restructure training and curricula for school nurses and preservice teacher education to systematically support digital content development.

While the participants in this study were generally resistant to the full development of metaverse-based content, they expressed strong enthusiasm for utilising and adapting pre-developed programmes in their classes. In this context, providing shared template-based frameworks that allow for contextual customisation will be a more practical and effective alternative. Such partially customisable templates can help reduce the burden of content creation while allowing teachers to integrate their professional judgement and expertise. Ultimately, this approach may enhance both the educational utility and real-world applicability of metaverse programmes in school settings.

There are several limitations that must be acknowledged. First, the study was conducted with a group of in-service school nurses from a specific region (City Y), which limits the generalisability of the findings to other geographic areas or school types. Second, the participants' experiences and perspectives were based on a single, short-term exposure to the programme. Therefore, the long-term impact of the MBST and its effectiveness when applied repeatedly in real educational settings were not fully captured. Third, the features and structure of the metaverse platform used in this study were based on a specific development environment. It is possible that the technical capabilities and user interface of the platform influenced participants' perceptions and experiences.

## Conclusions

The findings of this study indicate that the MBST holds promise as an innovative alternative for infectious disease preparedness education. This approach was perceived by the school nurses as an engaging and effective approach to infectious disease preparedness, promoting immersive and self-directed learning. However, for successful real-world application, structural barriers such as technological limitations, lack of institutional support, and content development burdens must be addressed. Emphasis was placed on school leaders cultivating a positive culture to enhance infrastructure and support systems.

### Clinical implications

The school nurses expressed a strong willingness to apply MBST in their workplaces. In order to support their will and ensure its effectiveness and sustainability, the following structural supports are essential: (1) user-friendly platform design and instructional manuals that consider accessibility for all staff involved; (2) strengthened collaboration across school communities and leadership; (3) professional development programmes focused on enhancing school nurses' capacity to design and modify digital educational content; and (4) customisable template structures that enable teachers to apply their professional expertise while reducing content development burdens.

## Data Availability

The data supporting the findings of this study are not publicly accessible in order to protect participant confidentiality and adhere to ethical guidelines. Relevant data for this study are available upon request from the corresponding author.
